# Supplementary material for: Combination of Blood Adiponectin and Leptin Levels Is a Predictor of Biochemical Recurrence in Prostate Cancer Invading the Surrounding Adipose Tissue
Source: Int J Mol Sci. 2024 Aug 17;25(16):8970. doi: 10.3390/ijms25168970 (PMC11354761; doi:10.3390/ijms25168970)
Supplement: Supplementary file 1 [file ijms-25-08970-s001.zip › ijms-3134396-supplementary.pdf]

Supplementary Materials

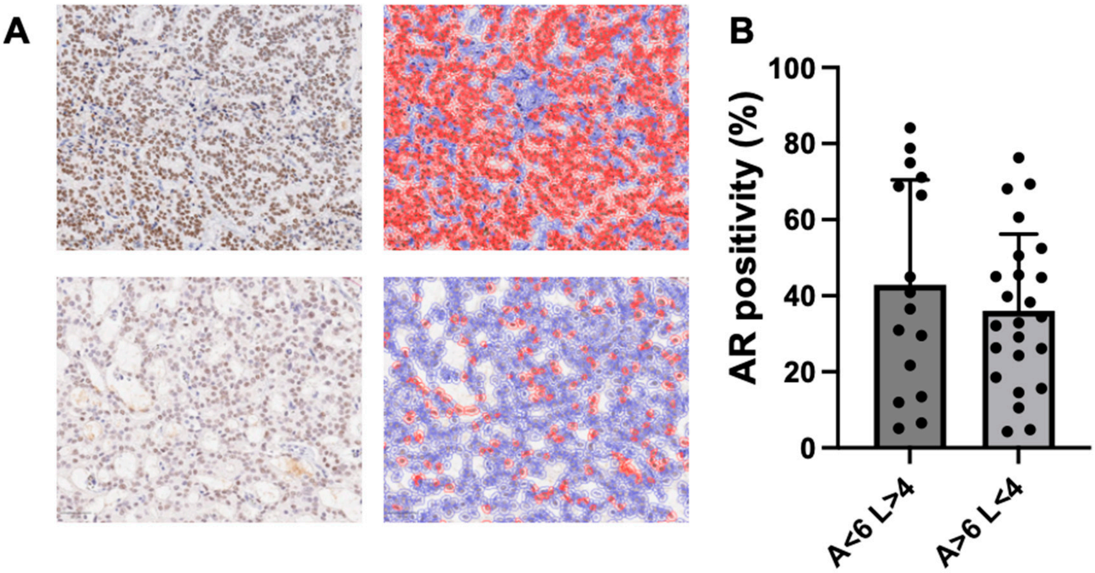

Supplementary Figure 1. Androgen receptor (AR) expression in prostate cancer tissue. (A) AR immunostaining and evaluation of level of positivity. Upper left, prostate cancer tissue with high AR-positivity. Upper right, positive cells (stained red) and negative cells (stained blue). Lower left, prostate cancer tissue with low AR-positivity. Lower right, positive cells (stained red) and negative cells (stained blue). Scale bar: 50 µm. (B) Statistical analysis of AR positivity between different adipokine concentration groups. "A<6, L>4" indicates mean adiponectin (<6 µg/ml) and leptin (>4 ng/ml) levels. "A>6, L<4" indicates mean adiponectin (>6 µg/ml) and leptin (<4 ng/ml) levels. Statistical analysis was performed using the unpaired t-test.

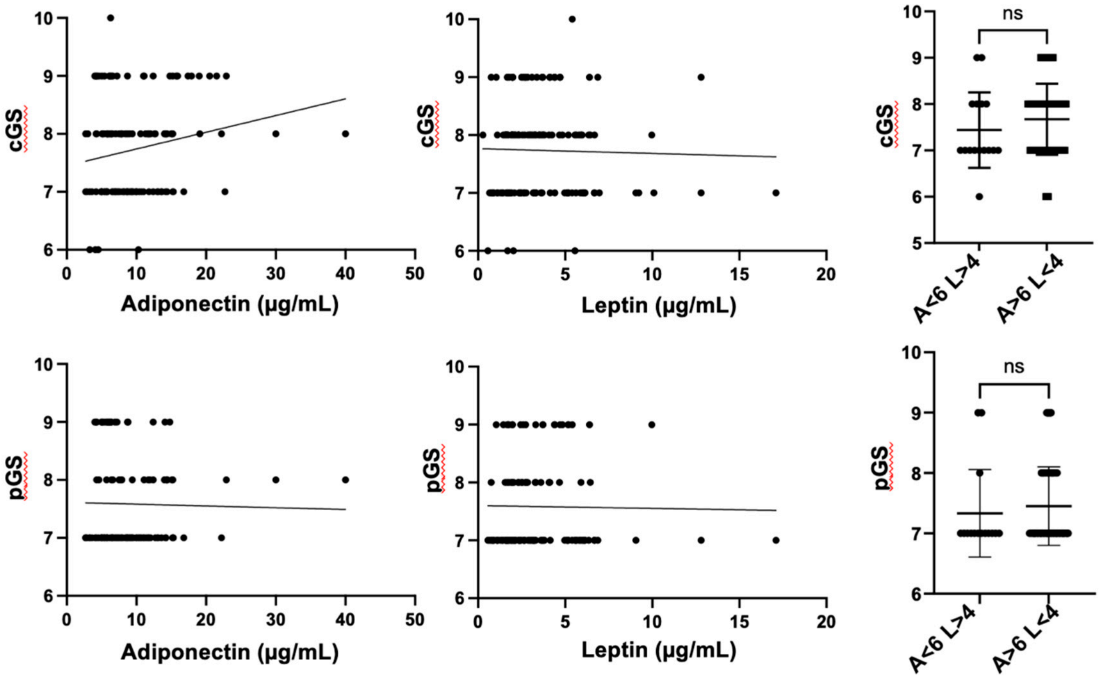

Supplementary Figure 2. Correlation between clinical (preoperative) or pathological (post-operative) Gleason scores and adipokine levels. cGS: clinical Gleason score, pGS; pathological Gleason score. Upper left, upper middle: Correlation between clinical or pathological Gleason scores and adipokine levels. Upper right: Statistical analysis of AR positivity between different adipokine concentration groups. “A<6, L>4” indicates mean adiponectin (<6 μg/ml) and leptin (>4 ng/ml) levels. “A>6, L<4” indicates mean adiponectin (>6 μg/ml) and leptin (<4 ng/ml) levels. Statistical analysis was performed using the unpaired t-test. Lower left, lower middle: Correlation between clinical or pathological Gleason scores and adipokine levels. Lower right: Statistical analysis of AR positivity between different adipokine concentration groups. “A<6, L>4” indicates mean adiponectin (<6 μg/ml) and leptin (>4 ng/ml) levels. “A>6, L<4” indicates mean adiponectin (>6 μg/ml) and leptin (<4 ng/ml) levels. Statistical analysis was performed using the unpaired t-test.
